# 6G Networks and the AI Revolution—Exploring Technologies, Applications, and Emerging Challenges

**DOI:** 10.3390/s24061888

**Published:** 2024-03-15

**Authors:** Robin Chataut, Mary Nankya, Robert Akl

**Affiliations:** 1 School of Computing and Engineering, Quinnipiac University, Hamden, CT 06518, USA; 2 Department of Computer Science, Fitchburg State University, Fitchburg, MA 01420, USA; mnankya@fitchburgstate.edu; 3 Department of Computer Science and Engineering, University of North Texas, Denton, TX 76203, USA; robert.akl@unt.edu

**Keywords:** 6G, 5G, artificial intelligence, machine learning, terahertz communication, ultra-massive MIMO, quantum communication, millimeter waves, blockchain, Internet of Things

## Abstract

In the rapidly evolving landscape of wireless communication, each successive generation of networks has achieved significant technological leaps, profoundly transforming the way we connect and interact. From the analog simplicity of 1G to the digital prowess of 5G, the journey of mobile networks has been marked by constant innovation and escalating demands for faster, more reliable, and more efficient communication systems. As 5G becomes a global reality, laying the foundation for an interconnected world, the quest for even more advanced networks leads us to the threshold of the sixth-generation (6G) era. This paper presents a hierarchical exploration of 6G networks, poised at the forefront of the next revolution in wireless technology. This study delves into the technological advancements that underpin the need for 6G, examining its key features, benefits, and key enabling technologies. We dissect the intricacies of cutting-edge innovations like terahertz communication, ultra-massive MIMO, artificial intelligence (AI), machine learning (ML), quantum communication, and reconfigurable intelligent surfaces. Through a meticulous analysis, we evaluate the strengths, weaknesses, and state-of-the-art research in these areas, offering a wider view of the current progress and potential applications of 6G networks. Central to our discussion is the transformative role of AI in shaping the future of 6G networks. By integrating AI and ML, 6G networks are expected to offer unprecedented capabilities, from enhanced mobile broadband to groundbreaking applications in areas like smart cities and autonomous systems. This integration heralds a new era of intelligent, self-optimizing networks that promise to redefine the parameters of connectivity and digital interaction. We also address critical challenges in the deployment of 6G, from technological hurdles to regulatory concerns, providing a holistic assessment of potential barriers. By highlighting the interplay between 6G and AI technologies, this study maps out the current landscape and lights the path forward in this rapidly evolving domain. This paper aims to be a cornerstone resource, providing essential insights, addressing unresolved research questions, and stimulating further investigation into the multifaceted realm of 6G networks. By highlighting the synergy between 6G and AI technologies, we aim to illuminate the path forward in this rapidly evolving field.

## 1. Introduction

As globalization advances, the volume of mobile data traffic is experiencing a rapid and exponential increase. According to a report by the ITU-R, global mobile data traffic was 158 exabytes per month in 2022 and is projected to reach 2194 exabytes per month by 2028 and 5016 exabytes per month by 2030 [1].These numbers represent an exponential increase in the amount of data consumed by mobile subscribers, with each subscriber projected to consume 257 gigabytes of data in 2030 compared to 12.1 gigabytes in 2022 [2]. The growing demand for mobile data services is not limited to a particular region or demographic [3]. By 2025, around 70% of the global population will utilize mobile services, with approximately 60% accessing mobile internet. This growth is further propelled by the proliferation of new technologies such as the Internet of Things, AI, blockchain, augmented and extended reality, 3D video, and connected vehicles [4].

5G technology has been deployed worldwide [5] to meet the increasing need for mobile data services. However, with the world moving towards automation, it is apparent that a more advanced technology than current 5G networks will be required to handle the rising data traffic [6]. This is where the sixth generation ‘6G’ network comes in, which is expected to provide users with high-quality service while coping with this exponential increment in data traffic [7,8]. The sixth-generation network promises to be a game-changer in mobile wireless technology, with its ultra-fast data speeds, low latency, and massive connectivity. 6G networks will transform mobile networks by integrating AI and ML to seamlessly combine the physical, digital, and biological worlds. This integration will enable the creation of new use cases and applications that were not previously possible with 5G networks. Moreover, 6G networks will lay the foundation for developing smart cities, autonomous vehicles, and other applications that require reliable, high-bandwidth, and low-latency connectivity [9]. In this paper, we take a hierarchical approach to 6G networks and present a comprehensive overview of the 6G networks. Our summary of contributions and paper organization is as follows.

**Figure 1:**
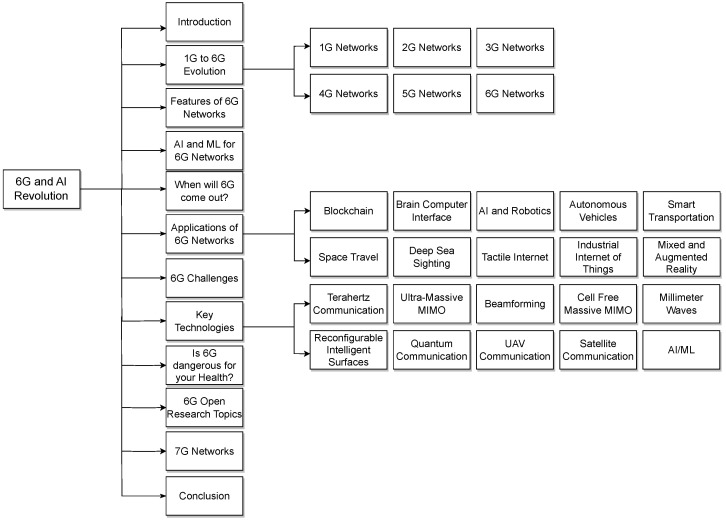
Organizational structure of the paper.

Section 2 delves into the technological foundations of 6G networks and the AI revolution, spanning from the inception of 1G to the cutting-edge developments of 6G and AI-revolutionized 6G networks and their anticipated impact on various sectors. In Section 3, we explore the distinctive features that define 6G networks, shedding light on the anticipated capabilities and innovations that set them apart from their predecessors. Section 4 discusses the integration of AI and ML in 6G networks. Section 5 addresses the pressing question of “When Will 6G Come Out?” by examining current timelines, ongoing research initiatives, and industry expectations surrounding the deployment of 6G technology. Section 6 discusses the convergence of AI, 6G, and wireless communication, highlighting enhanced mobile broadband (eMBB), ultra-reliable low-latency communication (URLLC), and massive machine-type communication (mMTC) use cases investigating the diverse applications of 6G networks, envisioning the transformative impact on industries, services, and everyday life. Section 7 scrutinizes the challenges associated with the deployment of 6G, ranging from technological hurdles to regulatory considerations, providing a comprehensive assessment of potential obstacles. In Section 8, we explore the key technologies that shape the deployment of the 6G network. Section 9 examines whether 6G poses health risks, delving into existing research surrounding the potential dangers of advanced wireless technologies and AI involvement in the mentioned key enabling technologies. Section 10 opens the door to future research endeavors by outlining unique recommendations and future research directions. Section 11 briefly touches upon the speculative realm of 7G networks, contemplating the potential directions and features that may define the next frontier in wireless communication. Finally, Section 12 concludes the comprehensive analysis, summarizing key findings and insights derived from the exploration of 6G networks and setting the stage for further research and development in the field. Figure 1 below shows the organizational structure for this paper.

## 2. Technological Foundations of 6G Networks and AI Revolution

The era of mobile communication started in the early 1980s and has seen significant development and expansion in the decades that followed. The advancement of mobile wireless technology can be divided into distinct eras, each of which has brought about substantial progress and developments in data rates, connectivity, and functionality [10]. The initial phase of mobile wireless technology, 1G, was introduced in the early 1980s and was primarily based on analog technology [11]. This generation of technology was primarily utilized for voice communication and was distinguished by its low data transfer speeds and subpar audio quality [12]. Some examples of 1G include Advanced Mobile Phone System (AMPS), Total Access Communication System (TACS), and Nordic Mobile Telephone (NMT) [13].

The introduction of second-generation (2G) mobile networks in the early 1990s marked a shift from analog to digital technology [14]. Along with traditional voice services, 2G networks introduced new capabilities such as short message service (SMS) and basic email functionality. 2G networks also improved audio quality and enhanced security [15]. Some of the well-known 2G (second generation) mobile networks include GSM (Global System for Mobile Communications), IS-95 (Interim Standard-95) [16] PDC (Personal Digital Cellular), and CDMAone (Code Division Multiple Access) [17].

Next was the introduction of 3G (third generation) mobile networks in the early 2000s, which marked a significant advancement in mobile technology, providing both voice and data services [18]. These networks offered elevated data transfer speeds and the capability of web browsing on mobile devices. They also introduced multimedia message support (MMS) and the ability to use data-intensive applications such as email, web browsing, video streaming, and mobile television [19]. In addition to providing enhanced data transfer speeds and web browsing capabilities, 3G networks expanded the coverage area and incorporated security measures such as packet data confidentiality and integrity. Some examples of 3G (third generation) mobile networks include CDMA2000 (Code Division Multiple Access 2000), WCDMA (Wideband Code Division Multiple Access), and EDGE (Enhanced Data rates for GSM Evolution) [20].

The 4G (fourth generation) mobile networks in the early 2010s marked a significant advancement in mobile technology, offering high data transfer speeds and improved network coverage [21]. These networks enabled HD video streaming, mobile video conferencing, online gaming, and high-speed mobile internet. Examples of 4G (fourth generation) mobile networks include LTE (Long-Term Evolution) and WiMAX (Worldwide Interoperability for Microwave Access) [22].

The introduction of 5G (fifth generation) mobile networks in the early 2010s represents the latest advancement in mobile technology, with the first 5G mobile towers coming online in 2018 [23]. These networks are distinguished by extremely high data transfer speeds, improved network coverage, and ultra-low latency. 5G networks are expected to be a foundation for the Internet of Things (IoT), smart cities, and the fourth industrial revolution [24].

While previous generations of wireless networks have already leveraged AI for optimization and automation, 6G takes this collaboration to unprecedented levels, integrating AI at every layer of the network architecture.

**Figure 2:**
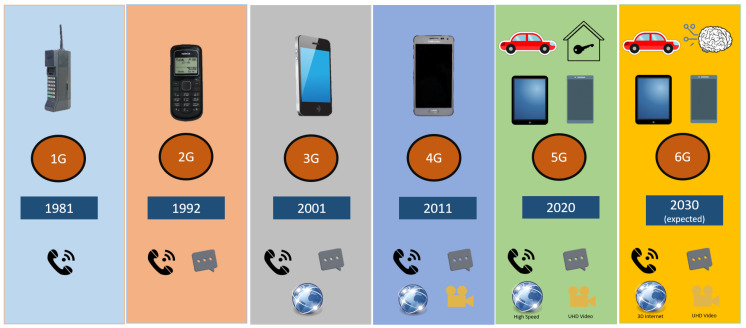
Evolution of mobile communications: a chronological depiction of the advancements in mobile network technology from 1G in 1981 to the expected 6G in 2030. This visual encapsulates the major milestones in mobile communications, including the emergence of 2G and the introduction of SMS in 1992, the advent of 3G and mobile data in 2001, the expansion to 4G and high-speed internet access in 2011, and the integration of IoT with 5G in 2020. The future projection of 6G suggests a paradigm shift to smarter, AI-driven networks supporting 3D internet and enhanced video capabilities.

6G networks are currently being researched and developed as the next evolution of mobile networks, with the expectation of providing unparalleled transmission speeds, ultra-low latency, and improved coverage [25]. These networks will incorporate cutting-edge technologies such as terahertz communication, ultra-massive MIMO, AI, machine learning (ML), quantum communication, millimeter, reconfigurable intelligent surfaces, etc. Potential applications for 6G networks include Linked robotic and self-governing systems, wireless brain–computer interfaces, blockchain advancements, immersive multi-sensory realities, space and deep-sea exploration, tactile internet capabilities, and industrial networking. Figure 2 shows the evolution of mobile communications.

### AI-Revolutionized 6G Networks and Their Anticipated Impact on Various Sectors

The convergence of 6G networks and AI technologies holds immense promise for transforming various sectors and unlocking new opportunities for innovation, efficiency, and economic growth. By harnessing the synergies between these two transformative technologies, we stand on the brink of a new era of connectivity, where the boundaries between the physical and digital worlds blur and the possibilities are limited only by our imagination. 6G is envisioned as a paradigm shift, introducing revolutionary capabilities that will reshape industries, economies, and societies at large, as seen below:

- **Healthcare:** 6G networks, coupled with AI, are poised to revolutionize healthcare delivery [26]. From remote patient monitoring to real-time telemedicine consultations, the ultra-low latency and high reliability of 6G will enable life-saving applications, such as remote surgery performed by robotic systems guided by AI algorithms. Furthermore, AI-driven predictive analytics can enhance disease detection and treatment outcomes, leading to more personalized and efficient healthcare services [27].
- **Transportation:** In the transportation sector, 6G networks will underpin the proliferation of autonomous vehicles and smart transportation systems [28]. AI-powered algorithms will leverage real-time data from vehicle-to-everything (V2X) communication to optimize traffic flow, enhance safety, and reduce congestion. Moreover, 6G’s ultra-reliable low-latency communication (URLLC) capabilities will enable split-second decision making, ensuring the seamless operation of autonomous vehicles even in the most demanding scenarios.
- **Manufacturing:** With the rise of Industry 4.0, 6G networks will catalyze the transition towards smart factories characterized by interconnected, AI-driven production systems. By leveraging 6G’s massive connectivity and AI’s predictive maintenance capabilities, manufacturers can optimize production processes, minimize downtime, and maximize resource efficiency [29]. Collaborative robots equipped with AI algorithms will enable agile and flexible manufacturing workflows, adapting in real time to changing demand and market dynamics.
- **Entertainment and media:** The convergence of 6G networks and AI will revolutionize the entertainment and media landscape, ushering in immersive experiences and personalized content delivery. From augmented reality (AR) and virtual reality (VR) applications to AI-generated content recommendations, 6G networks will enable seamless, high-fidelity multimedia streaming with minimal latency [30]. AI algorithms will analyze user preferences and behavior in real time, delivering hyper-personalized content tailored to individual tastes and preferences.
- **Finance and banking:** In the financial sector, 6G networks will facilitate the proliferation of AI-driven fintech solutions, revolutionizing payment systems, fraud detection, and risk management. Real-time data analytics powered by AI will enable financial institutions to detect fraudulent transactions with unprecedented accuracy. At the same time, 6G’s ultra-low latency will ensure near-instantaneous transaction processing, enhancing the efficiency and security of digital payments [31].

## 3. Features of 6G

**Figure 3:**
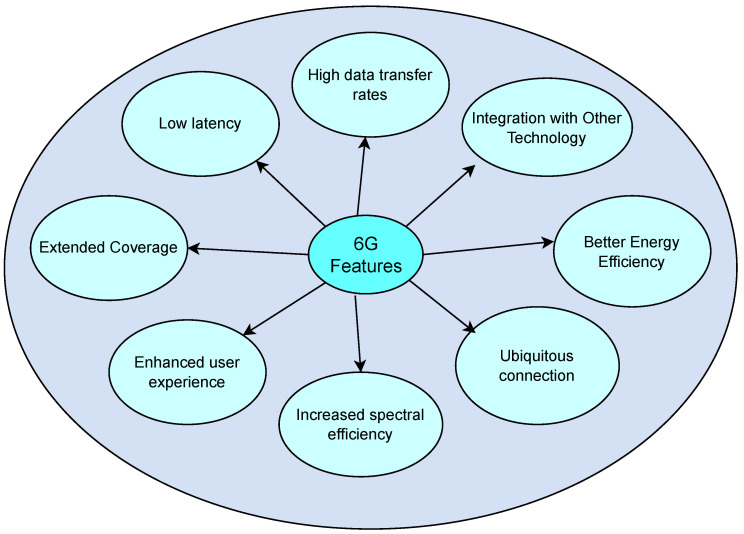
Features of 6G network.

6G networks are expected to bring significant advancements over the current 5G technology. The features and advantages of 6G networks are summarized in Figure 3. Here are some of the key features and potential advancements associated with 6G:

- **High data transfer rates**: 6G networks are expected to bring tremendous advancements in data transfer speeds, potentially reaching up to 10 Tbps. This represents a significant increase when compared to the current data transfer speed set for 5G networks, which is 10 Gbps [32].
- **Low latency:** 6G networks are expected to provide ultra-low latency, potentially reaching as low as 0.1 ms, which significantly improves upon the latency of 5G networks with a latency requirement of 1 ms [33].
- **Extended coverage:** 6G networks are expected to have an extended coverage range, potentially reaching deep-sea, space, and underground areas. This would enable the use of new applications such as deep-sea sightseeing, space travel, and industrial internet [34].
- **Enhanced user experience:** 6G networks are projected to enhance the user experience by amplifying the capabilities of extended reality, augmented reality, virtual reality, and AI [35].
- **Increased spectral efficiency:** 6G networks are expected to offer spectral and network efficiency ten times greater than that of 5G networks [36].
- **Ubiquitous connection:** 6G networks are expected to provide enormous broadcasting data and to support more than 1 million connections, which is a hundred times more than current 5G networks [37].
- **Better energy efficiency:** 6G networks are expected to have an optimized energy consumption, resulting in longer battery life, making it more sustainable and efficient to use [38].
- **Integration with other technology:** Anticipated integration of 6G networks involves seamless incorporation with other technologies, such as IoT, cloud computing, and big data analytics, ensuring efficient connections across various systems [39].

Figure 3 shows features of 6G network.

Table 1 shows the performance comparison of 4G, 5G, and 6G networks [40].

## 4. AI and ML for 6G Networks

AI and ML are anticipated to have a revolutionary impact on 6G networks, improving network management optimization and enhancing the user experience across various aspects. AI is expected to play a crucial role in developing 6G networks by addressing the primary challenge of managing the significant increase in connected devices and data traffic [41]. By optimizing network resources like bandwidth and computing power, AI can guarantee the efficient operation of the network [42]. AI can enable real-time decision-making capabilities, essential for applications like autonomous vehicles and augmented reality to operate safely and efficiently. Furthermore, AI can create intelligent networks that can self-learn and adapt in real time by analyzing data to improve performance and efficiency. It can predict potential issues by analyzing sensor data and other sources, preventing service disruptions. This predictive maintenance for 6G networks reduces downtime and increases reliability [43]. On the other hand, ML algorithms can analyze user behavior patterns and preferences to personalize the network experience. This includes adaptive content delivery, predictive caching, and personalized service recommendations. AI-driven cybersecurity measures can continuously analyze network traffic patterns, detect anomalies, and proactively respond to security threats [44]. ML models can evolve and adapt to new cyber threats, enhancing overall network security. AI can also be applied to cognitive radio systems, allowing networks to autonomously adapt to changing radio conditions, interference, and spectrum availability [45]. This enables the more flexible and intelligent use of available radio resources [46]. Moreover, AI-driven techniques can enhance the resilience of 6G networks by predicting and mitigating the impact of faults or disruptions. ML models can adaptively reroute traffic and optimize network performance during failures [47].

ML will play a crucial role in enabling the development of 6G networks by providing intelligent and adaptive capabilities that can support various applications and services [48]. These algorithms can dynamically manage and optimize network resources in real time, depending on the network’s specific use case and requirements. Reinforcement learning (RL) algorithms, such as Q-learning and Deep Q Networks (DQNs), can make sequential decisions in dynamic environments. In network management, RL can be applied to optimize resource allocation, routing, and scheduling based on changing conditions [49]. A multi-objective-based genetic algorithm is an optimization algorithm inspired by natural selection that can be used to solve resource allocation problems in networks, adapting to changing demands and constraints [50]. Particle swarm optimization (PSO) is a population-based optimization algorithm that models the social behavior of particles. It can be employed for dynamic resource allocation, load balancing, and network optimization [51]. Deep learning models like neural networks can undergo training to forecast network performance, recognize patterns, and enhance resource allocation [52]. Deep reinforcement learning (DRL) combines deep learning with RL for complex decision making. Fuzzy logic can be applied to model and control network parameters in a dynamic environment. It provides a way to handle uncertainty and imprecise information in network optimization [53]. Markov decision processes (MDPs) are used in RL to model decision-making problems with sequential interactions. In network optimization, MDPs can represent the dynamic nature of resource allocation and routing decisions [54]. Swarm intelligence algorithms inspired by swarm intelligence, such as the bee algorithm or the firefly algorithm, can be applied to optimize network resources collaboratively [55].

## 5. When Will 6G Come Out?

Anticipations suggest that 6G networks could debut around 2030, potentially emerging earlier in specific global regions. 6G wireless technology is currently the focus of research and development by several countries, universities, and tech companies worldwide. China has set an ambitious goal of dominating the 6G industry by 2030, and companies like Huawei, ZTE, and China Mobile are actively involved in 6G research [56,57,58,59]. In South Korea, LG has established a 6G research center [60], while Finland’s University of Oulu is leading 6G research with the country’s “6G Flagship” program [61]. The US Federal Communications Commission (FCC) has opened the “terahertz wave” for experiments on next-generation standards, which could include 6G. At the same time, companies like Qualcomm and Intel are also involved in 6G research [62]. Japan’s 6G research program focuses on developing technology for super-fast data transfer rates, and the EU’s Horizon 2020 5G-DRIVE project explores the potential of 6G [63,64]. However, despite the significant efforts being made, 6G technology is still in its early stages of development, and it may be some time before 6G networks become a reality.

## 6. The Convergence of AI, 6G, and Wireless Communication

With 6G technology, a wide array of possibilities unfolds across three key services: enhanced mobile broadband (eMBB), ultra-reliable low-latency communication (URLLC), and massive machine-type communication (mMTC) [65]. The above use cases are described in detail below:

(a) **Enhanced mobile broadband (eMBB):** 6G networks are expected to further improve upon the enhanced mobile broadband capabilities of 5G by delivering even higher data rates, lower latency, and increased capacity [66]. Some potential aspects of eMBB in 6G networks include the following:
  - Ultra-high data rates: 6G networks could achieve significantly higher data rates compared to 5G, potentially reaching terabits per second (Tbps) speeds [67]. This would enable seamless streaming of immersive, high-resolution content such as holographic videos, uncompressed 8K or 16K video streaming, and ultra-HD virtual reality (VR) and augmented reality (AR) experiences.
  - Low-latency communication: 6G networks aim to further reduce latency to near-real-time levels, enabling ultra-responsive applications such as cloud gaming, remote surgery, and autonomous vehicles. With latency reduced to microseconds or even nanoseconds, users can experience seamless interactions with remote systems and devices [68].
  - Massive capacity: 6G networks are expected to support a massive increase in connected devices and simultaneous connections, facilitating the proliferation of IoT devices, wearable technologies, and smart sensors [69]. This would enable seamless connectivity and data exchange in densely populated areas or scenarios with a high density of connected devices.
(b) **Ultra-reliable low-latency communication (URLLC):** 6G networks will build upon the ultra-reliable low-latency communication capabilities of 5G by further reducing latency and increasing reliability. Key aspects of URLLC in 6G networks include the following:
  - Mission-critical applications: 6G networks will support mission-critical applications that require ultra-low latency and high reliability, such as industrial automation, remote surgery, and autonomous vehicles. By reducing latency to sub-millisecond levels and ensuring ultra-reliable communication links, 6G networks will enable seamless connectivity and real-time responsiveness in critical scenarios [70].
  - Predictive maintenance: With advanced analytics and AI integration, 6G networks can enable predictive maintenance in industrial settings, allowing machines and equipment to communicate in real time and anticipate maintenance needs before failures occur. This proactive approach to maintenance can minimize downtime, reduce operational costs, and optimize asset performance [71].
(c) **Massive machine-type communication (mMTC):** 6G networks will continue to support massive machine-type communication, catering to the connectivity needs of many IoT devices and sensors. Key aspects of mMTC in 6G networks include the following:
  - Massive scalability: 6G networks will be designed to support a massive scale of connected devices, ranging from billions to trillions of IoT devices and sensors [72]. This scalability will enable the deployment of IoT solutions in various domains, including smart cities, industrial automation, agriculture, healthcare, and environmental monitoring.
  - Energy-efficient communication: 6G networks will incorporate energy-efficient communication protocols and techniques to optimize power consumption in IoT devices and extend battery life [73]. This will enable long-lasting and sustainable IoT deployments, particularly in remote or inaccessible locations where power sources are limited.
  - Diverse use cases: 6G networks will support diverse IoT applications with varying requirements in terms of data rates, latency, reliability, and energy consumption [74]. These applications include smart grids, asset tracking, environmental monitoring, smart agriculture, and smart healthcare, among others.

Table 2 compares 5G and AI-revolutionized 6G technology across enhanced mobile broadband, ultra-reliable low-latency communication, and massive machine-type communication.

### 6.1. Applications of 6G Network

The 6G network is not yet commercially available; however, it is expected to have applications in several domains [81].

**Figure 4:**
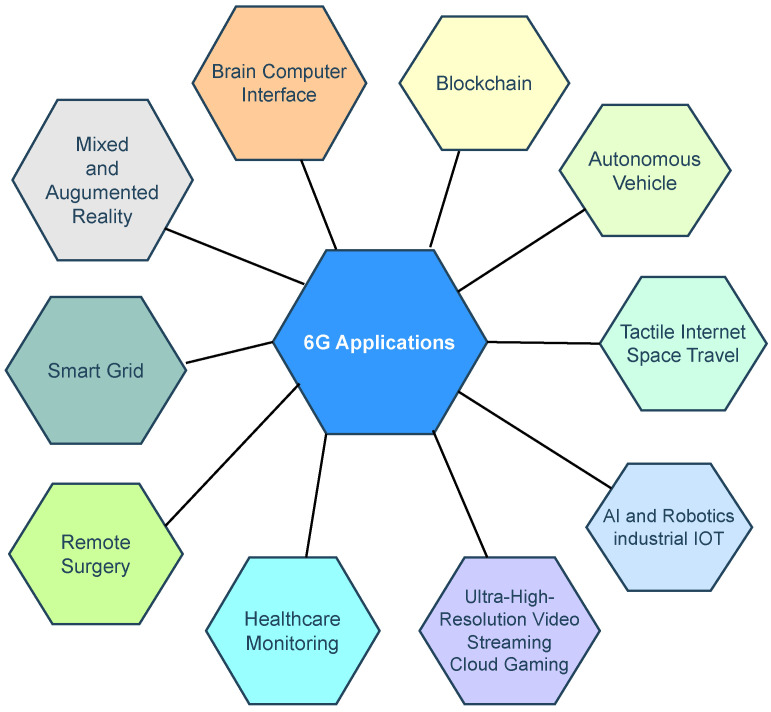
Applications of 6G networks.

Figure 4 below shows the applications of 6G networks.

#### 6.1.1. Ultra-High-Resolution Video Streaming and Cloud Gaming

The advancement of the 6G network and the AI revolution will enable seamless streaming of ultra-high-resolution video content and cloud gaming experiences with unprecedented quality and minimal interruptions [82]. Complementing this infrastructure, the AI revolution will leverage sophisticated algorithms to optimize streaming parameters in real time, ensuring smooth playback and personalized content recommendations based on user preferences. Together, this integration of 6G networks and AI technologies will revolutionize the entertainment landscape, offering users immersive, high-quality experiences that redefine the boundaries of mobile communication and AI.

#### 6.1.2. Healthcare Monitoring

The convergence of AI and the 6G revolution is poised to revolutionize health monitoring applications by enabling real-time data processing, remote monitoring, personalized healthcare, predictive analytics, healthcare automation, and enhanced diagnosis and treatment [83]. The ultra-low latency and high data transfer speeds offered by 6G networks will facilitate the seamless transmission and analysis of health data, allowing for timely interventions and personalized healthcare recommendations based on individualized data insights. AI algorithms will be crucial in analyzing vast datasets to identify patterns and correlations, enabling predictive analytics for forecasting health trends and automating various healthcare processes [84]. This synergy between AI and 6G technologies promises to optimize patient care by providing more efficient, proactive, and personalized healthcare delivery.

#### 6.1.3. Remote Surgery

The emergence of telesurgery, enabled by robotic technology and wireless networking, revolutionizes surgical practice by connecting geographically distant patients and surgeons [85]. This innovative approach harnesses the power of advanced telecommunications and robotics, facilitated by the anticipated 6G network revolution and artificial intelligence technologies. The 6G network’s ultra-fast data transmission speeds and ultra-low latency ensure seamless communication between remote robotic surgical systems and surgeons, overcoming geographical barriers and providing high-quality surgical care in remote locations [86]. AI-powered robotic systems exhibit precise and dexterous movements, augmented by AI algorithms that continuously learn and adapt to improve surgical outcomes. Real-time data processing and analysis by AI algorithms enable remote surgeons to make informed decisions during telesurgery procedures, enhancing patient safety and surgical precision. Overall, integrating 6G networking capabilities and AI technologies in telesurgery advances surgical practice by improving access to care, enhancing surgical outcomes, and ensuring patient safety.

#### 6.1.4. Smart Grid

Due to the integration of 6G and artificial intelligence, a significant revolution is underway in the development and functionality of smart grids. 6G networks are anticipated to offer ultra-fast data transmission speeds, ultra-low latency, and extensive device connectivity, thereby significantly improving the communication infrastructure of smart grids [87]. This will enable the real-time monitoring, control, and optimization of energy distribution and consumption within the grid, facilitating efficient energy management and response to dynamic demand patterns. AI technologies, when integrated with 6G networks, further enhance the capabilities of smart grids by enabling advanced analytics, predictive modeling, and autonomous decision making. AI algorithms can analyze vast amounts of data collected from sensors, meters, and other grid components to identify patterns, predict potential issues, and optimize grid operations in real time [88]. This synergy between 6G and AI revolutionizes the efficiency, reliability, and resilience of smart grids, paving the way for a more sustainable and intelligent energy infrastructure.

#### 6.1.5. Brain–Computer Interfaces

Brain–computer interfaces (BCIs) aim to establish a direct link between the brain and a computer, enabling individuals to manipulate machines through their thoughts [89]. Unlike traditional input devices, such as a mouse or keyboard, a BCI decodes and interprets brain signals and converts them into control commands that the computer can execute. The objective of BCIs is to empower individuals to control machines with their thoughts alone, for instance, to operate a prosthetic limb or a wheelchair.

With the emergence of 6G, BCIs could potentially benefit from advancements in communication technologies [90]. 6G networks are projected to offer higher data transfer rates and shorter latencies, making it possible to process brain signals in real time. This is crucial for the efficacy of BCIs, as real-time processing and analysis of brain signals are vital. The new technologies, such as terahertz communication and edge computing, available with 6G can potentially lead to the creation of advanced, compact BCI devices with improved precision and reliability. Furthermore, integrating BCIs with other cutting-edge technologies, such as IoT and AI, could offer new avenues for developing BCI applications, including context-aware and personalized BCI-based solutions. It is essential to note that although 6G holds excellent promise for BCIs, much research and development is still required to realize these possibilities fully.

#### 6.1.6. Blockchain

Blockchain is a system that allows for the secure and transparent recording of digital transactions in a decentralized manner [91]. Integrating 6G networks with blockchain technology offers a range of benefits and potential applications. 6G, with its projected enhancements in data transfer rates, lower latency, and increased network capacity, could support the real-time processing of complex transactions and applications.

One potential use of blockchain technology in 6G is to improve security and privacy in communication and transactions [92]. The increased speed and capacity of 6G networks may enable the implementation of blockchain-based solutions to enhance data security and protect against cyberattacks and data breaches. Another application is the development of decentralized platforms and networks by integrating blockchain technology with 6G. 6G’s enhanced speed and capacity could support the creation of decentralized networks that securely store and manage large amounts of sensitive data, such as financial transactions or medical records. These decentralized systems may offer improved security and privacy compared to centralized systems and could drive the development of new services and applications. Although 6G is still in its early stages of development, the potential applications of blockchain technology in 6G are vast and have the potential to significantly change the way we communicate, store, and manage data.

#### 6.1.7. Space Travel

Space exploration is an area that could greatly benefit from the advancements in 6G technology. With its improved speed and capacity, 6G could facilitate real-time communication between spacecraft and ground control, streamlining missions and enabling agile decision making [93]. The fast data transfer speeds offered by 6G allow the effective transfer of substantial volumes of remote sensing data, leading to more accurate and detailed information and the potential for new scientific discoveries. Furthermore, the strong and secure communication networks enabled by 6G could connect spacecraft and ground control, ensuring reliable and uninterrupted communication. Additionally, 6G’s enhanced network capacity and low latency could support the transmission of high-resolution virtual and augmented reality data, offering an improved immersive experience for those involved in space exploration.

#### 6.1.8. Deep-Sea Sightseeing

Applying 6G in deep-sea sightseeing could enhance the underwater experience for individuals. With 6G’s increased data transfer rates and reduced latency, real-time communication between the deep sea and the surface could be established [94]. This could allow for the transmission of high-quality images, videos, and data from the ocean’s depths in real time, providing a more immersive experience for deep-sea observers. Additionally, 6G’s improved network capacity and increased speed could support the deployment of underwater drones and other autonomous vehicles for deep-sea exploration [95]. These vehicles could be equipped with high-resolution cameras and other sensing devices to collect and transmit data, enabling the collection of more accurate and detailed information about the deep-sea environment. The implementation of 6G in deep-sea sightseeing holds great promise, but much research and development work is still needed to realize its full potential.

#### 6.1.9. Tactile Internet

The tactile internet is an emerging field that seeks to create a new form of human–machine interaction through the sense of touch [96]. Applying 6G technology to the tactile internet could significantly enhance its capabilities and potential applications. 6G makes it possible for real-time, high-fidelity transmission of touch-based data. With 6G, it may be possible to create more advanced and responsive haptic systems that can provide a realistic simulation of touch, allowing for remote control and manipulation of objects, including virtual and augmented reality applications. 6G could also provide the high-speed and low-latency connectivity required to support the real-time teleoperation of robots and other remote-controlled devices, allowing for more precise and effective control. Additionally, 6G’s advanced communication technologies, such as edge computing and terahertz communication, may enable the development of compact and highly accurate haptic devices [97]. Integrating 6G technology into the tactile internet could open up new possibilities for human–machine interaction and have far-reaching implications for healthcare, gaming, and manufacturing industries.

#### 6.1.10. Industrial Internet of Things

The combination of 6G and the potential for transformation lies within the Industrial Internet of Things (IIoT) industrial operations [98]. 6G’s real-time communication capabilities can result in more agile and efficient processes. Additionally, 6G’s high data processing speeds can lead to improved decision making and increased accuracy in industrial processes. 6G’s advanced security features can better protect against cyber threats and data breaches in industrial settings. Integrating 6G and IIoT can also open the door to new IoT-based solutions, such as the predictive maintenance and remote control of industrial systems [99]. With its ability to drive the creation of intelligent and automated industrial systems, 6G has the potential to increase productivity and efficiency and to lower costs.

#### 6.1.11. Mixed and Augmented Reality

6G technology presents an incredible opportunity to enhance mixed and augmented reality (MAR) experiences. 6G’s real-time capabilities enable the seamless merging of virtual and physical realms, providing users with a more immersive and interactive experience [100]. The transmission of high-resolution virtual and augmented reality data enabled by 6G can provide improved visual and sensory experiences in MAR applications. This opens up new possibilities for education, entertainment, and product visualization. Additionally, 6G’s ability to connect individuals in virtual environments can lead to new ways for remote work, social interaction, and gaming to occur. 6G’s enhanced network security measures can provide peace of mind, protecting sensitive information and user data from cyber threats [101]. The merging of 6G and MAR technology has significant potential to generate inventive and immersive experiences, establishing it as a primary application of 6G technology.

#### 6.1.12. AI and Robotics

The application of 6G on AI and robotics is expected to be significant and impactful due to the increased capabilities and improved connectivity of 6G networks [102]. With 6G, AI algorithms will see a boost in accuracy and speed, while autonomous robots and drones will be equipped with real-time communication and control features. Advanced AI-powered systems, such as self-driving vehicles, smart factories, and intelligent homes, will become more sophisticated. With increased natural language processing abilities and a more comprehensive range of applications, virtual assistants will also improve. 6G will enable the remote control and monitoring of AI and robotic systems in hazardous environments, and AI will be used for predictive maintenance and monitoring in industrial settings. The increased connectivity and capabilities of 6G networks will also drive the creation of new and innovative AI-powered applications and services.

#### 6.1.13. Autonomous Vehicles and Smart Transportation Systems

The application of 6G technology in autonomous vehicles and smart transportation systems is poised to bring significant advancements and improvements [103]. 6G networks will offer the vital infrastructure for the secure and effective functioning of autonomous vehicles, facilitating real-time communication and control among vehicles, the central traffic management system, and the surrounding infrastructure [104]. The deployment of 6G will augment the safety and dependability of autonomous vehicles through more rapid and precise decision making. Furthermore, real-time data exchange between vehicles and infrastructure will optimize traffic management and flow, increasing efficiency and reducing congestion. The superior connectivity and features of 6G networks will foster the growth of cutting-edge smart transportation systems and services while also advancing existing autonomous vehicle technologies, such as sensors and mapping capabilities [105].

#### 6.1.14. Mission-Critical Services (MCSs)

6G networks offer a transformative platform for enhancing mission-critical services (MCSs) through ultra-reliable low-latency communication, real-time monitoring and control of critical infrastructure, integration with edge computing and AI for predictive analytics and decision making, advanced public safety and emergency response applications, development of smart infrastructure and utilities for improved efficiency and resilience, support for telemedicine and remote healthcare services, and robust cybersecurity measures to protect sensitive data and infrastructure from cyber threats. These capabilities enable timely decision making, seamless coordination among emergency services, enhanced reliability of essential services, and improved accessibility to critical care, ultimately ensuring MCSs’ reliability, responsiveness, and efficiency across various sectors [106].

#### 6.1.15. Public Protection and Disaster Relief (PPDR)

The advent of 6G networks presents many transformative applications for public protection and disaster relief (PPDR) efforts. Through ultra-high-speed, low-latency communication, 6G facilitates the real-time data transmission essential for swift decision making and coordination among emergency responders during crises [107]. Integration with augmented reality (AR) and virtual reality (VR) technologies enhances situational awareness and navigation in disaster zones. At the same time, AI-powered predictive analytics enables proactive PPDR strategies by analyzing vast datasets to anticipate risks and optimize resource allocation. Seamless integration with drones and UAVs enables aerial surveillance and search-and-rescue missions, while biometric identification and wearable technologies ensure the safety and accountability of personnel in disaster areas. Advanced security measures safeguard sensitive PPDR data and communication infrastructure, while community engagement platforms empower citizens to participate actively in disaster preparedness and response efforts. Overall, 6G networks hold immense potential to enhance the effectiveness, efficiency, and resilience of PPDR initiatives, ultimately saving lives and mitigating the impact of disasters on communities [108].

## 7. Challenges for 6G Deployment

**Figure 5:**
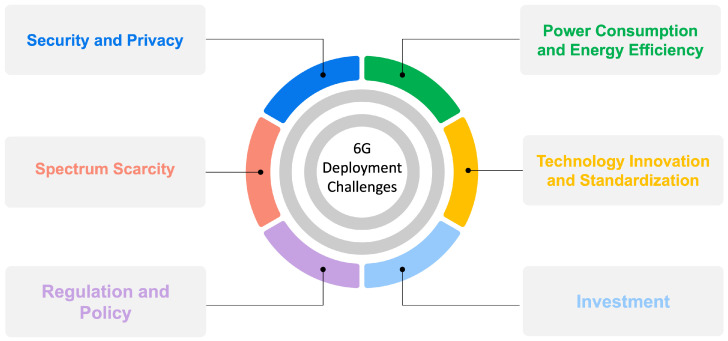
Challenges for 6G deployment.

The deployment of 6G technology faces numerous deployment challenges. Some of them are discussed in this section (Figure 5).

- **Technology innovation and standardization:** Technical difficulties are a challenge in implementing new enabling technologies like millimeter- and terahertz-wave communication, massive and ultra-massive MIMO, AI, ML, quantum communication, and ultra-reliable low-latency communication [109].
- **Bandwidth scarcity:** Identifying and allocating sufficient spectrum in the Terahertz (THz) frequency range for 6G is a significant challenge. THz frequencies offer the potential for high data rates but come with propagation challenges and require new regulatory frameworks [110].
- **Interoperability with existing networks:** Ensuring interoperability between different technologies across various industries and use cases is a complex challenge as many other networks use different standards and protocols [111].
- **Investment cost:** The deployment of 6G infrastructure is expected to be cost-intensive, requiring substantial investments in advanced technologies, equipment, and infrastructure. This might pose a financial challenge for network operators and end-users. This financial burden could hinder the broad adoption of 6G, especially in less economically developed regions and remote rural areas [112].
- **Regulation and policy:** Regulatory issues may arise due to new spectra and technologies, necessitating developing and implementing new policies and regulations [113].
- **Power consumption:** Power consumption is another concern, as the increased data rates and the number of devices connected to the network will result in higher power usage. Sharing a spectrum and infrastructure, implementing cell-free massive MIMO, and integrating communication and sensing are all pivotal aspects. Yet, the paramount transformation with 6G lies in the shift to higher frequencies, surpassing the 100 GHz threshold [114].
- **International collaboration and harmonization:** The competitive landscape, with multiple companies and countries vying to be the first to launch and deploy 6G, is a challenge. Promoting collaboration and harmonization of 6G standards and regulations on a global scale is crucial to ensure the success and widespread adoption of 6G technology and will be challenging.
- **Security and privacy:** There will be new security concerns as the network will transmit large amounts of sensitive data. Besides increasing connectivity and integrating various devices and systems, security and privacy will be a significant challenge [115].
- **Environmental concerns:** The production of 6G infrastructure requires various raw materials, including rare earth metals and minerals. The extraction processes can have environmental and social impacts, contributing to habitat destruction, pollution, and resource depletion [116].

Table 3 summarizes the 6G deployment challenges and possible solutions.

## 8. Key Technologies for 6G Deployment

**Figure 6:**
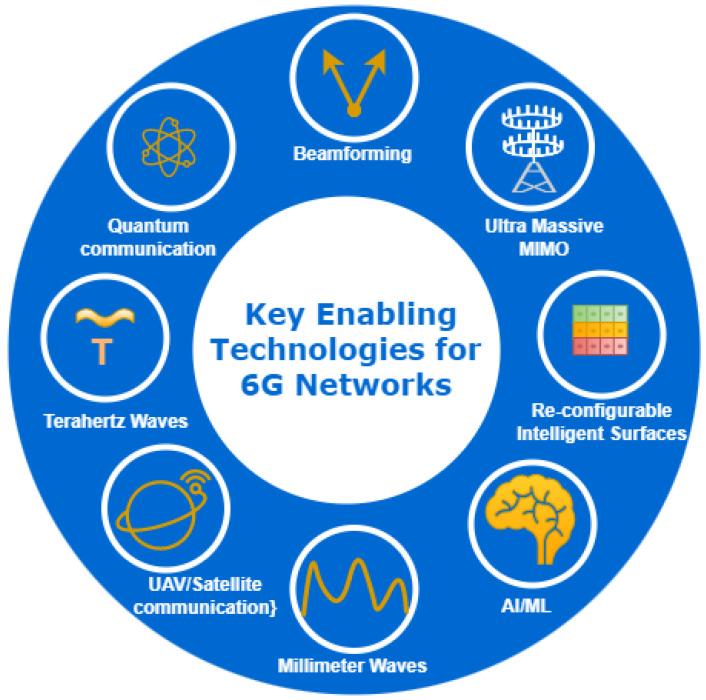
Overview of key enabling technologies for 6G networks. This diagram illustrates the advanced technological pillars essential for the deployment of 6G networks, including quantum communication, beamforming, ultra-massive MIMO, reconfigurable intelligent surfaces, AI/ML, millimeter waves, UAV or satellite communication, and terahertz waves. Each technology is crucial for enhancing future wireless communication systems data rate, reliability, and overall efficiency.

Several key technologies are being explored and considered as potential components of 6G networks as shown in Figure 6. We will discuss a few major enabling technologies for 6G networks.

### 8.1. Terahertz Communication

Terahertz communication is expected to significantly impact the development of 6G networks by offering faster and more efficient data transmission capabilities [135]. Terahertz communication provides higher data rates than current wireless communication technologies, resulting in more immediate download and upload speeds and improving the overall user experience. Moreover, terahertz frequencies provide more available bandwidth, allowing for more efficient spectrum use, reducing network congestion, and improving overall network performance [136]. Terahertz communication can enable new use cases, such as high-resolution imaging, remote sensing, and advanced medical imaging, which require higher data rates and lower latency [137]. Using short wavelengths that are difficult to intercept or detect, terahertz communication can enhance wireless communication security, reducing the risk of cyber attacks and unauthorized access [138].

### 8.2. Ultra-Massive MIMO

Ultra-massive MIMO technology, a key component in the evolution of 6G networks, offers significant advancements in network capacity, data rates, and coverage. It utilizes an extensive array of antennas capable of transmitting and receiving multiple data streams simultaneously. This capability not only accelerates data transmission but also significantly boosts data rates [139]. Furthermore, ultra-massive MIMO enhances signal processing and improves beamforming, leading to higher energy efficiency and more effective spectrum utilization.

This technology also plays a crucial role in optimizing frequency spectrum use, augmenting network capacity, and enhancing coverage. Its ability to reduce interference substantially improves overall network performance. Additionally, ultra-massive MIMO is instrumental in supporting emerging applications that demand higher data rates and lower latency. These applications include virtual reality, autonomous vehicles, and the development of smart city infrastructure [140]. Consequently, ultra-massive MIMO is a transformative technology poised to revolutionize 6G network capabilities and facilitate a new wave of technological advancements.

### 8.3. Beamforming

Beamforming is a crucial technology that improves the efficiency and reliability of wireless communication and enables the development of 6G networks. This technology involves directing radio waves in a specific direction to achieve better spectrum use and network performance. In 6G networks, beamforming can focus wireless signals on the desired receiver, resulting in reduced interference and improved signal strength [141]. This leads to higher data rates, lower latency, and the ability to support real-time data transmission for new use cases like virtual reality, remote surgery, and autonomous vehicles. Beamforming technology enables the more efficient use of the frequency spectrum by directing radio waves to specific areas [142]. This decreases interference and enhances network capacity, mitigating congestion and improving overall performance.

### 8.4. Cell-Free Massive MIMO

Cell-free massive MIMO is a promising technology for the development of 6G networks, as highlighted in [143]. This technology involves deploying numerous antennas across a given area, enhancing the efficiency of wireless communication. Compared to traditional cellular networks, cell-free massive MIMO offers several advantages, including improved network coverage. This is particularly beneficial in dense urban environments, where it allows for the more effective use of available radio resources, leading to better signal quality and fewer coverage gaps [144]. Cell-free massive MIMO technology leverages AI for resource allocation, interference coordination, and user scheduling to maximize network capacity and mitigate interference.

Additionally, cell-free massive MIMO can support a more significant number of users per unit area, thus increasing network capacity. This feature is especially useful in areas with high user density, such as stadiums, airports, and other public spaces [145]. Another significant advantage of cell-free massive MIMO is the reduction in latency. By enabling multiple users to access the same channel simultaneously, it reduces waiting times and improves the overall user experience [146].

Furthermore, cell-free massive MIMO contributes to enhanced energy efficiency. Reducing the need for complex and power-intensive signal processing algorithms leads to lower power consumption and extended battery life for mobile devices [147]. These benefits make Cell-free Massive MIMO a transformative technology for future cellular networks.

### 8.5. Millimeter Waves

Millimeter waves (mmWaves) operate within a frequency range of 30 GHz to 300 GHz and have shorter wavelengths than the traditional microwave bands used in 4G and 5G networks [148]. Their potential to deliver faster data speeds, higher network capacity, and improved network efficiency make them a crucial enabler of 6G networks. MmWave technology provides several benefits, such as enabling high data rates of several gigabits per second and allowing for new use cases, such as augmented reality and 8K video streaming, that require high data rates and low latency. mmWave can increase network capacity as the higher frequencies make more efficient use of the available spectrum. AI algorithms dynamically adjust transmission parameters and predict channel blockage events, ensuring reliable connectivity and maximizing data rates in millimeter-wave communication.

However, the use of mmWave technology also poses challenges. One such challenge is the shorter range of mmWave signals compared to traditional microwave frequencies, making it easy for obstacles such as buildings and trees to block signals and affect network coverage [149]. Furthermore, deploying mmWave technology requires many antennas, resulting in high infrastructure costs that must be addressed to promote widespread technology adoption.

### 8.6. Reconfigurable Intelligent Surfaces

Reconfigurable intelligent surfaces (RISs) comprise an emerging technology with significant potential to drive the development of 6G networks. Characterized by a flat surface embedded with numerous small antennas or reflectors, a RIS can electronically control radio waves to reflect, amplify, or absorb them [150]. This capability offers several advantages for future network infrastructures.

One of the primary benefits of RIS technology is its ability to enhance network coverage. Placing RISs in areas with traditionally poor coverage or high signal interference, such as indoor spaces, can effectively reflect and amplify signals. This improvement results in stronger signal strength and broader coverage. Additionally, RIS technology contributes to increased network capacity by enabling more efficient use of available radio resources. It accomplishes this by focusing radio waves in specific directions, reducing interference and supporting more concurrent users.

Reconfigurable intelligent surfaces (RISs) are optimized by AI algorithms for efficient signal reflection and amplification, enhancing network coverage and capacity. AI-driven surface configurations adapt to changing environmental conditions and user demands, optimizing energy efficiency and spectral utilization for improved network performance.

Another significant advantage of RIS technology is its contribution to energy efficiency. By reflecting and focusing radio waves directionally, a RIS minimizes the energy required for transmitting signals over long distances [151]. This leads to lower power consumption, prolonged battery life for mobile devices, and a reduction in the overall energy footprint of the network. The widespread implementation of RIS technology will necessitate substantial investments in infrastructure and technological advancements. Addressing these requirements is essential to fully leverage the capabilities of RISs in enhancing future network systems.

### 8.7. Quantum Communication

Quantum communication is an advanced technology that can be utilized in the development of 6G networks [152]. Unlike traditional communication technologies that rely on electromagnetic waves to transmit data, quantum communication uses photons for transmission. This feature allows quantum communication to offer high levels of security, making it ideal for military and government communications where the highest levels of security are required.

Quantum communication can also support new applications that necessitate real-time data transmission, such as autonomous vehicles and smart cities. By offering instantaneous communication over long distances, quantum communication can reduce latency and enable faster response times [153]. However, deploying this technology faces several challenges, such as the need for specialized hardware and infrastructure and high implementation costs.

### 8.8. UAV/Satellite Communication

UAV/satellite communication is a technology that can facilitate the development of 6G networks [154]. This technology uses unmanned aerial vehicles (UAVs) and satellites to provide wireless connectivity to remote and underserved areas. By using these aerial platforms, it is possible to provide high-speed data transfer and internet connectivity to regions that are difficult to reach using traditional terrestrial networks. There is potential for expanded network coverage, particularly in remote and rural areas with limited conventional infrastructure. This can enable more people to access high-speed internet and other data services, improving access to information and enabling new applications and services. UAV/satellite communication can also support new use cases that require real-time data transmission, such as remote medical procedures and disaster response [155]. By enabling communication over long distances and in rugged terrain, UAV/satellite communication can improve the efficiency and effectiveness of these applications. The deployment of UAV/satellite communication technology also presents challenges, such as the need for specialized hardware and infrastructure, as well as regulatory issues related to the use of airspace [156]. The technology requires significant satellite and UAV deployment and maintenance investment.

Table 4 shows the role of AI in the above-discussed key technologies for 6G deployment.

## 9. Is 6G Dangerous for Your Health?

Research on the potential health effects of 6G networks is limited, as the technology is still in its early stages of development. However, concerns have been raised about the possible risks of exposure to high-frequency electromagnetic radiation in 6G networks [165]. The World Health Organization has categorized electromagnetic radiation as a potential carcinogen, and specific research studies have associated exposure to high levels of electromagnetic radiation with an elevated likelihood of cancer and other health issues [166]. However, these studies have focused mainly on exposure to radiofrequency radiation from cell phones and other devices that operate in lower-frequency ranges used by 4G and 5G networks [167]; while there is currently no evidence to suggest that exposure to the higher-frequency electromagnetic radiation used in 6G networks poses a significant health risk to humans, more research is needed to fully understand this technology’s potential health effects. According to the FCC, the frequency range designated for 6G is between 95 GHz to 3THz. Despite being three to a thousand times higher than 5G’s frequency, these ranges are still considered safe as they are non-ionizing [168].

Exposure to electromagnetic radiation from 6G networks is likely to be significantly lower than that from other sources, as the technology will likely use a combination of different frequency ranges, including lower frequencies employed in earlier generations of mobile networks. Measures can also be taken to reduce exposure to electromagnetic radiation, for instance, restricting cell phone and wireless device usage, employing protective cases, and keeping devices away from the body when in use [169]; while there is currently no evidence to suggest that 6G networks are dangerous for human health, further research is needed to understand the potential risks of this technology.

## 10. Recommendations and Future Research Direction

- To support the establishment of dedicated testbeds to validate and maximize the performance of millimeter- and terahertz-wave communication in diverse environments, reinforcement learning algorithms can be deployed to optimize the configuration of testbed parameters like antenna placement, transmit power, and channel allocation. Further research is needed on new schemes adapted for the high-frequency bands and extensive bandwidths of 6G. This includes studying techniques for enhanced spectrum utilization and improved data throughput, critical for reliable communication in various environments, and training ML algorithms to process and analyze signals received from millimeter- and terahertz-wave communication systems. These algorithms can adaptively adjust parameters to optimize signal quality and mitigate interference in diverse environments.
- There is a need to design smart and intelligent protocols and architectures for efficient network handover and connectivity in remote areas exhibiting adaptability by seamlessly adjusting to changing network conditions, such as signal strength fluctuations and congestion. These systems should demonstrate context awareness by gathering and analyzing contextual data like location and user preferences to inform decision making. These systems should utilize predictive algorithms to anticipate handover events, enabling proactive connectivity and resource allocation optimization. With dynamic resource allocation, they can optimize network performance and efficiency by allocating bandwidth and spectrum based on real-time demands. Efficient routing algorithms should be utilized to minimize latency and maximize throughput during handover and connectivity establishment with intelligent decision-making mechanisms, including ML algorithms that can autonomously analyze network conditions to make optimized decisions regarding handover initiation and resource allocation.
- Natural language processing techniques can process unstructured network data, such as log files and network performance reports, to extract insights and identify potential congestion points or anomalies. AI-driven congestion management will dynamically monitor network traffic and identify congestion points, implementing strategies such as traffic shaping and rerouting to alleviate congestion and maintain optimal performance. Through predictive analytics, AI algorithms analyze historical network data to forecast future network behavior, enabling proactive resource allocation and capacity planning to prevent congestion and service degradation.
- Improvements to address the challenges posed by the increasing number of connected devices entail a two-fold approach: creating low-power hardware solutions and implementing sustainable network operation methods in the 6G network. Low-power hardware solutions involve designing energy-efficient components, optimizing circuit designs, employing low-power communication protocols, and integrating energy harvesting technologies like kinetic energy harvesters, piezoelectric materials, radio frequency energy harvesting, and thermoelectric generators into devices. Sustainable network operation methods encompass dynamic power management, resource sharing and allocation, deployment of green networking technologies, development of energy-aware routing algorithms, and implementation of life-cycle management practices.
- Invest in research and development of AI-driven encryption techniques, such as homomorphic encryption, which is a form of encryption that allows computations to be performed on encrypted data without decrypting them first. In other words, it enables operations such as addition and multiplication to be carried out on ciphertexts, resulting in the same operations being performed on the plaintexts when decrypted. This property is beneficial in scenarios where data privacy is crucial, as it allows for secure computation of sensitive data while keeping them encrypted throughout the process. For example, lattice-based cryptography utilizes the hardness of specific lattice problems, such as the shortest vector problem (SVP) or the learning with errors (LWE) problem, as the basis for cryptographic schemes. These problems are believed to be computationally difficult to solve, even for quantum computers, making lattice-based cryptography resistant to attacks from both classical and quantum adversaries. Post-quantum cryptography utilizes the hardness of specific lattice problems, such as the shortest vector problem (SVP) or the learning with errors (LWE) problem, as the basis for cryptographic schemes. These problems are believed to be computationally difficult to solve, even for quantum computers, making lattice-based cryptography resistant to attacks from classical and quantum adversaries. These techniques leverage AI algorithms to enhance data encryption security, enabling secure storage and transmission of sensitive information in interconnected environments, and can be improved with more advanced research.
- Research should focus on developing quantum key distribution schemes that are practical, scalable, and compatible with the high-speed and high-bandwidth requirements of 6G networks. Further investigation is needed into using entanglement as a resource for secure and efficient data transmission in 6G networks. Researchers should explore methods for generating, distributing, and utilizing entangled quantum states to enhance communication security and enable novel communication protocols with superior performance.
- Research in 6G networks should focus on leveraging their unique capabilities, including ultra-high data rates, ultra-reliable low-latency communication (URLLC), massive connectivity, high-precision positioning and navigation, edge computing, and network slicing, to enable transformative applications such as immersive virtual and augmented reality (VR/AR), autonomous vehicles, smart city infrastructure, digital twins, and edge computing. By exploring the potential of these capabilities and developing innovative solutions that harness their full potential, researchers can pave the way for a more connected, intelligent, and efficient future, where advanced applications and services enhance user experiences and drive societal and economic benefits.
- Researchers should focus on developing advanced network slicing in 6G networks that encompass architectures, algorithms, and management frameworks that enable the customization and efficient allocation of network resources for different services and applications. Hierarchical slicing architectures hierarchically organize network slices to facilitate resource sharing and isolation, while cloud-native slicing architectures leverage cloud-native principles for dynamic slice creation and deployment. Dynamic resource allocation algorithms dynamically allocate network resources among slices based on real-time demands, while QoS-aware routing algorithms determine optimal paths for traffic within slices. SDN-based and NFV-based slicing management frameworks facilitate centralized control and automated life-cycle management of network slices, enabling operators to efficiently tailor network services to diverse application requirements.
- Focusing on ultra-reliable low-latency communication (URLLC) in 6G networks is crucial for supporting critical applications such as remote healthcare and industrial automation. Ultra-reliable low-latency communication (URLLC) in 6G networks relies on specific standards, protocols, and technologies tailored to meet the stringent requirements of critical applications like remote healthcare and industrial automation. The 5G NR URLLC standard defined by 3GPP specifies parameters for achieving ultra-reliable and low-latency communication [170]. At the same time, IEEE 802.1 Time-Sensitive Networking (TSN) provides mechanisms for deterministic and low-latency communication in industrial automation. One-Pair Ethernet (OPE) offers cost-effective communication for automotive applications, and Time-Critical Networking (TCN) standards by the Industrial Internet Consortium ensure deterministic communication in industrial settings. Additionally, Non-Orthogonal Multiple Access (NOMA) is explored as a promising technique for efficient and reliable URLLC in dense networks. These standards, protocols, and technologies collectively enable ultra-reliable and low-latency communication, crucial for supporting critical services in 6G networks.

## 11. 7G Networks

7G networks, currently a theoretical concept, are anticipated to significantly outpace the capabilities of 6G networks, introducing groundbreaking features such as holographic communication and brain–computer interfaces. Presently, the term “7G” has not been officially recognized by any standardization bodies, and the realization of these networks is projected to span several decades. This timeline is due to the substantial advancements needed in wireless communication technology and infrastructure.

The vision for 7G includes the potential use of beyond-terahertz frequencies, which could dramatically increase data transmission speeds. Additionally, advancements in neuromorphic computing are expected to enhance data processing efficiency substantially. Among the other prospective features of 7G networks are highly sophisticated AI, seamless inter-network connectivity, and novel applications like fully autonomous transportation systems.

Despite the exciting prospects that 7G networks present, their development poses significant challenges. These include the need for extensive investment in research and development and considerable upgrades to existing communication infrastructure. Such developments are essential to leap from theoretical ideas to practical implementation, and this process is likely to span multiple decades.

## 12. Conclusions

This paper provides an in-depth exploration of the evolving landscape of 6G networks, highlighting the transformative role of artificial intelligence in this next wave of wireless technology. We presented the hierarchical structure of 6G networks, scrutinizing the technological advancements driving its emergence, the potential applications, and the challenges it faces. We explored the evolutionary trajectory of wireless communication technologies, emphasizing how each generation has progressively enhanced data rates, connectivity, and functionality. With the advent of 6G, we anticipate an unprecedented leap in these aspects, driven by key enabling technologies like terahertz communication, ultra-massive MIMO, AI, ML, quantum communication, and reconfigurable intelligent surfaces.

Integrating AI into 6G networks emerged as a pivotal theme, promising to revolutionize network management and user experience. By leveraging AI’s predictive and adaptive capabilities, 6G networks are poised to offer optimized bandwidth, enhanced efficiency, and more personalized services. The potential applications of 6G are vast and varied, ranging from enhanced mobile broadband to innovative domains such as smart cities, autonomous systems, and brain–computer interfaces, underlining the network’s transformative impact across sectors.

However, the journey towards actualizing 6G is not without its challenges. Technological hurdles, bandwidth scarcity, interoperability issues, investment costs, regulatory complexities, environmental concerns, and security and privacy issues represent significant barriers. Addressing these challenges requires a concerted effort from industry, academia, and regulatory bodies. As we venture into the speculative realm of 7G networks, we contemplate even more advanced features and capabilities. The prospect of holographic communication and neuromorphic computing in 7G underscores wireless technology’s continual evolution and boundless potential.

In conclusion, this paper serves as a comprehensive overview of 6G networks and a catalyst for further research and development. It underlines the synergy between 6G and AI technologies and sets the stage for continued exploration and innovation. As the world gravitates towards increasingly connected and intelligent systems, the insights and discussions presented here will be instrumental in shaping the future of wireless communication.

## Figures and Tables

**Table 1:** Comparison of 4G, 5G, and 6G performance indicators.

Performance Indicator	4G	5G	6G
Maximum data transfer rate	100 Mbps	10 Gbps	Up to 10 Tbps
Minimum latency	10 ms	1 ms	Up to 0.1 ms
Maximum device density per sq km	0.1 million devices	1 million devices	10 million devices
Energy efficiency	1×	100× more efficient than 4G	100× more efficient than 5G
Spectral efficiency	1×	100× more efficient than 4G	100× more efficient than 5G
Available spectrum	Up to 6 GHz	Up to 300 GHz	Up to 3 THz
Maximum mobility	200 km/h	300 km/h	600 km/h
AI integration	None	Partial	Full

**Table 2:** A comparison between 5G and AI revolutionized 6G technology across eMBB, URLLC, and mMTC.

Technology	Enhanced Mobile Broadband (eMBB)	Ultra-Reliable Low-Latency Communication (URLLC)	Massive Machine-Type Communication (mMTC)
5G	Provides high data rates for mobile users, enabling high-definition video streaming [75].	Offers reliable and low-latency communication, crucial for applications such as industrial automation and remote surgery [76].	Supports a large number of connected devices, enabling efficient communication between a massive number of IoT devices [77].
AI-revolutionized 6G	Leverages AI for better spectrum utilization and intelligent resource allocation [78].	Improves URLLC with even lower latency and higher reliability through AI-powered network optimization and predictive maintenance capabilities [79].	Optimizes resource allocation and communication protocols for efficient device-to-device communication and network slicing [80].

**Table 3:** Summary of challenges and possible solutions for 6G deployment.

Challenges	Possible Solutions
Technology innovation and standardization	Establish testbeds to validate the performance of millimeter- and terahertz-wave communication in different environments. This includes testing for signal propagation, interference, and device compatibility. Invest in developing signal-processing algorithms that can efficiently handle the massive number of antennas involved in MIMO systems. This includes beamforming, channel estimation, and interference management [117,118,119,120].
Scarcity of high-frequency spectra for bandwidth allocation	Collaborate with regulatory bodies to identify and allocate specific frequency bands for 6G, focusing on millimeter and terahertz bands. This involves conducting spectrum studies to identify underutilized or unallocated frequency ranges [121,122].
Create interoperability between current and 6G networks	The technologies should be built to interoperate with the existing network and devices [123,124].
Investment cost	Implement a phased approach to 6G deployment, focusing on specific geographic areas, use cases, or network functionalities. This approach minimizes high upfront costs [125].
Regulatory and policy challenges	Establish international agreements and collaborate with regulatory bodies to harmonize spectrum allocation for 6G. Encourage the development of dynamic spectrum-sharing technologies to optimize spectrum utilization [126].
Power consumption	A model for optimizing power has been introduced for a 6G-enabled massive IoT network. The primary objective is to enhance overall system performance, providing energy-saving features. Through efficient power resource management, the model minimizes power overhead attributed to the extensive number of connected devices. The proposed network assessment includes analyzing the maximum allocated power and spectral efficiency under various network operations and distinct precoding schemes [127,128].
International collaboration and harmonization	Encourage international collaboration in standardization bodies to develop unified standards for 6G technologies. Harmonize spectrum allocation, protocols, and interfaces to ensure interoperability and a consistent user experience [129].
Security and privacy	While robust security mechanisms are in place for safeguarding data during transit, there is a pressing need to prioritize data protection in processing and storage for comprehensive end-to-end security in 6G. Techniques such as oblivious computing, confidential computing, homomorphic encryption, and privacy-centric identifiers can be employed across 6G network services and components [130,131].
Environmental concerns	Design devices and infrastructure for longevity and ease of recycling. Establish collection and recycling programs for end-of-life electronic components. Encourage manufacturers to adopt sustainable product life cycles [132,133].
The complexity of AI integration	Since AI algorithms require large amounts of data and computational resources, which can strain network infrastructure and increase operational overhead, develop AI algorithms optimized for resource-constrained environments to reduce computational complexity. Implement edge computing and distributed AI architectures to offload processing tasks from centralized networks and invest in AI hardware accelerators and efficient algorithms to improve performance while minimizing resource consumption [134].

**Table 4:** The role of AI in the key technologies for 6G deployment.

Key Technologies for 6G Deployment	The Role of AI and ML in the Key Technologies
Terahertz communication	Terahertz communication in 6G networks promises faster and more efficient data transmission, aided by AI’s ability to optimize signal processing and security measures. Through real-time adaptation of transmission parameters, AI enhances network performance and mitigates signal attenuation, ensuring robust and secure wireless communication [157].
Ultra-massive MIMO	AI algorithms dynamically adjust antenna configurations and predict user demand patterns, maximizing spectral efficiency and user throughput. This synergy between AI and ultra-massive MIMO enhances network coverage and reliability while mitigating interference for improved quality of service [158].
Beamforming	AI-driven beamforming techniques enhance spectral efficiency and mitigate multi-path fading, ensuring seamless connectivity and reliability in dynamic urban environments [159].
Cell-free massive MIMO	Cell-free massive MIMO, characterized by deploying numerous antennas across an area to enhance wireless communication efficiency, leverage AI for various crucial tasks. AI algorithms are utilized for resource allocation, interference coordination, and user scheduling to maximize network capacity and mitigate interference effectively [160].
Millimeter waves	AI algorithms dynamically adjust transmission parameters and predict channel blockage events, ensuring reliable connectivity and maximizing data rates in millimeter-wave communication [161].
Reconfigurable intelligent surfaces	The role of AI in reconfigurable intelligent surfaces (RISs) is instrumental in optimizing their configuration for efficient signal reflection and amplification, thereby enhancing network coverage and capacity. AI algorithms adaptively adjust RIS configurations based on changing environmental conditions and user demands, optimizing energy efficiency and spectral utilization for improved network performance [162].
Quantum communication	Quantum communication in 6G networks is enhanced by AI’s optimization of quantum key distribution protocols and network management systems. AI algorithms optimize encoding strategies and automate key distribution tasks, ensuring secure and reliable data transmission over quantum communication channels [163].
UAV/satellite communication	UAV/satellite communication benefits from AI-driven optimization of trajectories and resource allocation, ensuring seamless connectivity in remote areas by predicting user demand patterns and dynamically allocating resources, enhancing network coverage and reliability for improved access to high-speed internet and data services [164].

## Abbreviations

The following abbreviations are used in this manuscript:

MDPI	Multidisciplinary Digital Publishing Institute
DOAJ	Directory of open access journals
TLA	Three-letter acronym
LD	Linear dichroism
AMPS	Advanced Mobile Phone System
TACS	Total Access Communication System
NMT	Nordic Mobile Telephone
SMS	Short message service
PDC	Personal Digital Cellular
WCDMA	Wideband Code Division Multiple Access
MMS	Multimedia message support
RL	Reinforcement learning
RIS	Reconfigurable intelligent surface
